# Synaptic Homeostasis and Its Immunological Disturbance in Neuromuscular Junction Disorders

**DOI:** 10.3390/ijms18040896

**Published:** 2017-04-24

**Authors:** Masaharu Takamori

**Affiliations:** Neurological Center, Kanazawa-Nishi Hospital, Kanazawa, Ishikawa 920-0025, Japan; m-takamori@vanilla.ocn.ne.jp; Tel.: +81-76-233-1811; Fax: +81-76-221-8603

**Keywords:** neuromuscular junction, agrin, Wnts, muscle-specific tyrosine kinase, low-density lipoprotein receptor-related protein 4, laminins, myasthenia gravis, nicotinic acetylcholine receptor, muscarinic acetylcholine receptor, adenosine receptor

## Abstract

In the neuromuscular junction, postsynaptic nicotinic acetylcholine receptor (nAChR) clustering, trans-synaptic communication and synaptic stabilization are modulated by the molecular mechanisms underlying synaptic plasticity. The synaptic functions are based presynaptically on the active zone architecture, synaptic vesicle proteins, Ca^2+^ channels and synaptic vesicle recycling. Postsynaptically, they are based on rapsyn-anchored nAChR clusters, localized sensitivity to ACh, and synaptic stabilization via linkage to the extracellular matrix so as to be precisely opposed to the nerve terminal. Focusing on neural agrin, Wnts, muscle-specific tyrosine kinase (a mediator of agrin and Wnts signalings and regulator of trans-synaptic communication), low-density lipoprotein receptor-related protein 4 (the receptor of agrin and Wnts and participant in retrograde signaling), laminin-network (including muscle-derived agrin), extracellular matrix proteins (participating in the synaptic stabilization) and presynaptic receptors (including muscarinic and adenosine receptors), we review the functional structures of the synapse by making reference to immunological pathogenecities in postsynaptic disease, myasthenia gravis. The synapse-related proteins including cortactin, coronin-6, caveolin-3, doublecortin, R-spondin 2, amyloid precursor family proteins, glia cell-derived neurotrophic factor and neurexins are also discussed in terms of their possible contribution to efficient synaptic transmission at the neuromuscular junction.

## 1. Introduction

The neuromuscular junction is a cholinergic synapse where agrin, Wnts, low-density lipoprotein receptor related-protein 4 (Lrp4), muscle-specific tyrosine kinase (MuSK) and extracellular matrix proteins are required for the complex differentiation and precise alignment of the pre- and postsynaptic structures. The signalings via agrin/Lrp4-MuSK (Ig1/2 domains, participating in the innervated stage of muscle) and Wnt/Lrp4-MuSK (cysteine-rich domain, participating in the non-innervated stage of muscle and axonal guidance) contribute to nicotinic acetylcholine receptor (nAChR) clustering, trans-synaptic communication and synaptic stability [1,2]. In myasthenia gravis (MG) mainly caused by the nAChR antibodies, a proportion of the patients harbor antibodies recognizing MuSK and Lrp4 which are responsible for pre- and postsynaptic impairments and contributive, at least in part, to a defect in ACh-release upregulation to compensate for postsynaptic dysfunction [3,4,5,6,7,8,9,10]. The following points will be emphasized below: (1) heterogeneity of postsynaptic MuSK and Lrp4 antibodies in their binding to functional domains which are responsible for pre- and postsynaptic functions; (2) the key molecular mediators such as Wnts, MuSK and Lrp4 act bidirectionally to form pre- and postsynaptic architectures; (3) the compensatory upregulation of ACh quantal release depending on the modulation via muscarinic and adenosine receoptors in the nerve terminal; and (4) Laminin-network and synaptic collagens (linking to MuSK) participating in synaptic stabilization.

## 2. Postsynaptic Organizations, Centered on MuSK, Lrp4 and Synapse-Related Proteins

**Figure 1:**
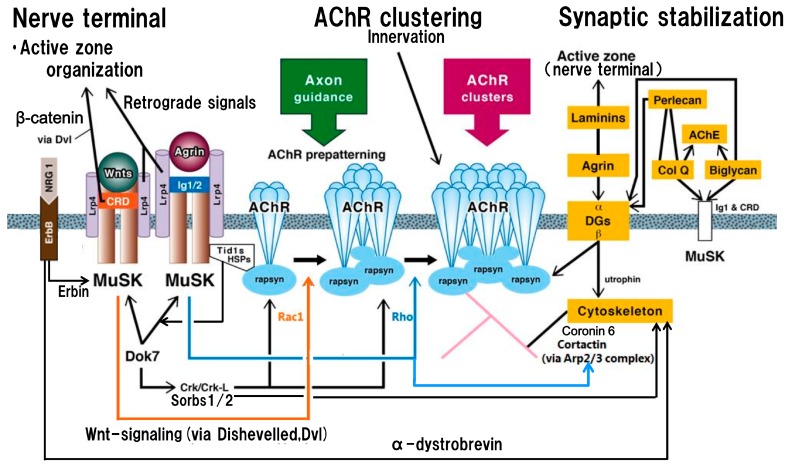
Schematic presentation of the postsynaptic structure and function on the basis of acetylcholine receptor clustering centered on agrin- and Wnt-signalings, trans-synaptic communication and synaptic stabilization. In the postsynaptic membrane, nicotinic acetylcholine receptors (nAChRs) are aggregated in the non-innervated stage via Wnts-MuSK cysteine-rich domain (CRD)-dishevelled protein signaling pathway to form nAChR microclusters (AChR prepatterning: located in the central part of the muscle membrane where incoming axons are guided) (orange line with arrow; via activation by Rac1), and in the innervated stage via agrin-MuSK immunoglobulin-like domains 1 and 2 (Ig1/2) signaling pathway to form full-sized nAChR clusters (blue line with arrow; via activation by Rho). The nAChR clusters in non-innervated and innervated stages are anchored by rapsyn (immobilized by heat-shock proteins, HSPs, including tumorous imaginal disc 1 short form, Tid1s, which belongs to HSP40 family) at the postsynaptic membrane. The kinase activity and subsequent downstream signalings by the intracellular tyrosine kinase domain of MuSK, located after the transmembrane segment, are crucial for the formation and maintenance of the neuromuscular junction. The low-density lipoprotein receptor-related protein 4 (Lrp4) plays an essential role in agrin- and Wnts-signaling pathways as the receptor for both signals. In the molecular structure of Lrp4, the first propeller domain interacts with agrin; in the third β-propeller domain, its edge part mediates the MuSK signaling and its central cavity mediates the Wnt signaling. As the trans-synaptic communication, the signaling mediated by Wnts-MuSK CRD contributes to the retrograde signal from muscle to nerve (Wnt canonical pathway via dishevelled protein (Dvl) and β-catenin), leading to presynaptic differentiation to localize active zone proteins for efficient synaptic transmission (left upper part). Others participating in the trans-synaptic communication are reviewed in detail in the text; among them, the retrograde signal of Lrp4 originates from its eight low-density lipoprotein a (LDLa) repeats to induce clustering of synaptic vesicle and active zone proteins [60] (left upper part). In addition, as shown in the right part (a part of synaptic stabilization), Laminins conduct the retrograde signal to firm the active zone architecture for sufficient synaptic transmission. Intracellularly, Dok7 (downstream of kinase 7) and neuregulin 1 (NGR1)-ErbB receptor (receptor tyrosine kinase of EGF, epidermal growth factor, receptor family) (mediator: Erbin) interaction activate intracellular MuSK tyrosine kinase domain for nAChR cluster formation (left part); also, they contribute to postsynaptic stability via respective downstream effectors. The mediator for Dok7 signal is Sorbs1/2 (downstream effectors of CT10 regulators of kinase (Crk/Crk-L)); the mediator for NGR1-ErbB receptor interaction is α-dystrobrevin (both are indicated by long black lines from left to right with arrows). As shown in the right part (yellow frames) of the figure, the stability of the neuromuscular junction including AChR clusters, MuSK and acetylcholinesterase (AChE) in the postsynaptic membrane is modulated by the extracellular matrix proteins (collagen Q (Col Q), perlecan, biglycan (glycosaminoglycan-binding form) and dystroglycans (DGs)), which participate in cytoskeletal dynamics. In addition, the postsynaptic structure is stabilized by the laminin-network including laminins, muscle agrin and DGs. The transmembrane dystroglycan (β-type) binds to rapsyn for anchoring AChR clusters at the postsynaptic membrane and also link utrophin to cytoskeleton for synaptic stability. Additionally, Col Q (C-terminus) and biglycan (non-glycanated form) bind both MuSK extracellular domains (Ig1 and CRD), leading to their implication in reinforcing a functional bridge between the agrin-signaling and the Wnt-signaling. Cortactin acts as a tyrosine kinase substrate and also a regulator of actin polymerization via actin-related proteins 2/3 complex (Arp2/3 complex); its tryrosine phosphorylation is enhanced by agrin/MuSK signaling (as indicated by blue line from central to right with arrow). Coronin 6 contributes to firm nAChR clustering via the modulation of actin dynamics.

MuSK is uniquely positioned as a key protein in the neuromuscular junction (NMJ). The ectodomain of muscle-derived MuSK consists of four immunoglobulin-like domains (Ig domains). The cysteine-rich domain (CRD, Ig4 domain) interacts with Wnts and thereby operates on synaptic function via the Wnts non-canonical signaling pathway [1,2,11,12,13,14,15,16,17,18]. Wnts belong to the Wingless-type mouse mammary tumor virus (MMTV) integration site family of glycoproteins which are released from motor neurons or derived from muscles; 19 different Wnt molecules exist in mice and humans [2]. The interaction of MuSK CRD with Wnts leads to nAChR clustering at the non-innervated stage of muscle through dishevelled scaffolding protein (Dvl) for the prepatterning of nAChR clusters (nAChR microcluster formation at the central part of muscle membrane where incoming axons are guided) and converges on the neural agrin-mediated signaling (Figure 1). The first and second immunoglobulin-like domains (Ig1/2 domains) conduct the neural agrin signal to form full-sized nAChR clusters in the innervated stage of muscle [1,2,19,20,21,22] (Figure 1). The nAChR clusters are anchored in the muscle membrane by rapsyn which is immobilized by MuSK-linking heat-shock proteins [23,24] (Figure 1). The negative regulation by the muscle-derived Wnt (such as Wnt3a)-canonical pathway via β-catenin/glycogen synthese kinase-3 reduces the expression of rapsyn, resulting in the reduction of agrin-mediated AChR clustering; this regulation maintains balance with the positive regulatory neuron-derived Wnt (such as Wnt3) and thereby helps to sculp the mature synaptic architecture; Wnt3 activates Rac1 in a more efficient usage than agrin which preferentially increases Rho activity [15,17]. The muscle-derived Lrp4 contributes to nAChR cluster formation by acting as the receptor for both Wnts and agrin at both non-innervated and innervated muscle membranes; Lrp4 can activate MuSK even without agrin [25,26,27,28,29] (Figure 1). In the molecular structure of Lrp4, the first propeller domain interacts with agrin [20]; in the third propeller domain, its edge part mediates the MuSK signaling and its central part mediates the Wnt signaling [30]. Adenomatous polyposis coli (APC) contributes to nAChR clustering and supports a cross-talk between agrin- and Wnt-mediated signaling pathways by coordinating the function of actin and the microtubule cytoskeleton during synapse formation [31].

The MuSK ectodomain also plays a role in the interaction of MuSK with matrix proteins [32,33,34,35,36], such as collagen Q/perlecan [37], biglycan [38,39] and cortactin [40], to contribute to postsynaptic stabilization in cooperation with laminin-network (including laminins α4, α5 and β2, muscle-derived agrin and dystroglycans which link to rapsyn and utrophin) [32,33,34,35,36,41,42,43,44] (the right yellow frames in Figure 1). The synaptic collagens (such as IV and XIII), nidogen-2 and rapsyn-interacting molecules (such as heat-shock proteins, α-actinin and calpain) are important for the maintenance of the neuromuscular junction [1,2,36]. Intracellularly, the interaction of neuregulin 1 with ErbB receptor (receptor tyrosine kinase of EGF, epidermal growth factor, receptor family) activates MuSK through the adaptor protein erbin to increase the tyrosine phosphorylation of MuSK and thereby modulate MuSK-dependent nAChR clustering [45,46] (Figure 1). In addition, the neuregulin 1-ErbB receptor interaction contributes to the stabilization of the postsynaptic apparatus through the phosphorylation of α-dystrobrevin [47] (Figure 1). In another intracellular signaling cascade, Dok-7 (downstream of kinase 7) forms a dimeric unit to dimerize and activate MuSK [48,49] and also recruits two adaptor proteins, Crk and Crk-L (CT10 regulators of kinase) which play an early role in the rapsyn-anchored nAChR cluster formation [50]. Downstream effects of Crk-L (Sorbs1/2) on cytoskeletal dynamics stabilize the postsynaptic organization [51] (Figure 1).

## 3. Trans-Synaptic Communication

The trans-synaptic communication is mediated via the Wnts canonical signaling by way of Wnts/MuSK CRD/Dvl/inhibition of glycogen synthese kinase 3β/β-catenin/Slit 2 in the muscle [52,53,54] (left upper part of Figure 1). This signaling leads to presynaptic differentiation to localize active zone proteins, synaptic vesicle proteins and Ca^2+^ channels at the nerve terminal, thereby conditioning the release-ready ACh-containing vesicles [55,56,57,58,59]. Besides the Wnts canonical signaling, muscle-derived Lrp4 interacts with an Lrp4-binding protein in motor neurons and acts as a retrograde signal to promote presynaptic differentiation [60,61] (left upper part of Figure 1). In addition, the muscle-derived synaptic organizers, laminins α4, α5 and β2, contribute to organization of the presynaptic active zone structure for ACh release [41,42,43,44] (right upper part of Figure 1). Laminin β2 tethers P/Q- and N-type voltage-gated Ca^2+^ channels (VGCCs) to the presynaptic active zone and cytoskeletal elements, thereby stabilizing the active zone structure for ACh release [41,42]. In the molecular structure of P/Q-type VGCC α1-subunit, laminin-binding domain [42] corresponds to the region that we determined the S5–S6 linker of domain III as a major focus both of the antibodies in patients with Lambert-Eaton myasthenic syndrome (LEMS, an autoimmune presynaptic disease caused mainly by antibodies against P/Q-type VGCC and partly by antibodies against synaptotagmin 1) and in the induction of LEMS animal model (active immunization model) [62].

Presynaptically, the α2δ-3 auxiliary subunit of the presynaptic Ca^2+^ channel is required for rapid homeostatic signaling which controls the active zone protein-dependent readily releasable ACh-containing vesicle pool [63]. Postsynaptically, in the innervated stage of muscle, ACh stimulates cyclin-dependent kinase 5 (postsynaptic serine/threonine kinase) to interact with nestin and thus inhibits the dispersion of AChR clusters which are not stabilized by agrin [1,64].

In view of these observations, particularly paying attention to the roles of MuSK in Wnts/MuSK CRD non-canonical and canonical pathways, we assayed the serum samples from 43 nAChR antibody-negative MG patients using the recombinant proteins expressed in HEK 293F cells as antigens [65]. The result showed that MuSK Ig1/2 antibodies were positive in 33 patients, 10 of whom (30%) were also positive for MuSK CRD antibodies (Table 1). The longitudinal epitope mapping study in 53 MuSK antibody-positive MG patients by the European research group showed that 22.6% of the patients were positive for MuSK CRD antibodies, although they emphasized the MuSK Ig1 domain as the main immunogenic region [66]. Although no patients were positive for MuSK CRD antibodies alone [65], the Japanese research group studied five MuSK antibody-positive patients and reported that the MuSK antibodies recognized the MuSK Ig1 domain and MuSK CRD in three patients and only MuSK CRD in two other patients [67]. It seems likely that the MuSK antibodies have heterogeneity in their binding to functional domains responsible for pre- and postsynaptic functions. To determine the functional significance of MuSK CRD (Frizzled-like domain) in the NMJ, the French research group showed that the CRD deletion of MuSK in mice caused exuberant axonal growth bypassing nAChR clusters and decreased synaptic vesicle density (presynaptic impairment), and a drastic deficit in nAChR clustering (postsynaptic impairment) [68]. They also suggested an implication of MuSK CRD in the Wnt-canonical signaling by demonstrating that these pre- and post-synaptic impairments were rescued by lithium chloride which acts as an inhibitor of the glycogen synthese kinase-3 and an activator of the Wnt/β-catenin signaling [68]. The New York research group and associates have recently proposed that dependently on species, the prepatterning of AChR clusters is influenced by an additional presence of kringle domain in the MuSK extracellular region [69]. The pathogenic variety of MuSK antibodies is further suggested by the fact that the collagen Q–MuSK interaction is blocked by MuSK antibodies [70], leading to hypersensitivity of the muscle membrane to ACh. This is based on the evidence that the synaptic anchorage of acetylcholinesterase (AChE) by collagen Q partly depends on the association of collagen Q with MuSK [37,71]. In fact, the MuSK MG animal models showed the electrophysiological finding characterized by slow miniature end-plate potential kinetics and hypersensitivity to AChE inhibitors [4,5]. In coordination with MuSK, the fetal nAChR γ-subunit [72] (antibodies to this subunit are detected in fetal nAChR inactivation syndrome, FARIS [73]), and the L-type Ca^2+^ channel dihydropyridine receptor [74] (antibodies to this receptor are detected in some MG patients [75]) are important in muscle prepatterning of AChR clusters which participate in axonal guidance to the muscle [1,2,14,17,29].

The electrophysiological study in MG with nAChR antibodies reported that compensatory ACh-release is upregulated but cannot be sustained at the high-frequency of nerve stimulation, possibly because of reduced pool of releasable ACh-containing vesicles [9,76]. In the nerve terminal, the homeostatic signal targets both Ca^2+^ influx and the release-ready vesicle pool [77]. During high frequency transmission, however, the early enhanced ACh quantal release causes the homeostatic upregulation of release based on Ca^2+^-dependent docking/priming of a small homeostatic reserve pool of vesicles (different from the pool of vesicles normally released); once the small pool of vesicles is depleted by the block of vesicle refilling, this homeostatic upregulation of ACh quantal release is no longer observed [78]. From the viewpoints of presynaptic autoreceptors, it should be taken into consideration that the mechanism underlying the interplay between presynaptic muscarinic and adenosine receptors controls ACh release in mammalian motor nerve terminals depending on the nerve stimulation paradigm; the A2A adenosine receptor (operating Ca^2+^ influx via L-type channels) plays a key role during long-lasting and/or high-frequency nerve activity [79,80,81,82,83,84], a situation that is impaired in toxin-induced myasthenia gravis and dysfunctional in experimental autoimmune myasthenia gravis [85,86].

## 4. Agrin, Cortaction and the Other Synapse-Related Proteins Contributing to the Modulation of Pre- and Postsynaptic Organizations

Agrin antibodies have been detected in MG patients including four triple negative MG patients (i.e., no detectable nAChR, MuSK or Lrp4 antibodies) [87,88]. The muscle agrin participates in postsynaptic stabilization as a protein included in laminin-network via its laminin-like G2 domain and α-dystroglycan. The neural agrin has amino acid insertion at two splicing sites and functions via its laminin-like G3 domain, thereby contributing to Lrp4/MuSK-mediated nAChR clustering at the postsynaptic membrane [21,89,90,91,92]. Besides the immunological implication, the dysfunction of agrin may be brought about by degradation due to the high level of matrix metalloproteinases (membrane-anchored extracellular proteases) [93] which was detected in sera of some MG patients [94,95]; this biological event could underlie muscle weakness in these reported patients. On the other hand, the expression of agrin in the nerve is upregulated by Brain-derived neurotrophic factor (BDNF)-Receptor tyrosine kinse B (TrkB) interaction which is brought about via Ca^2+^-response in the presynaptic Schwann cells containing transforming growth factor (TGF)-β1, thereby promoting agrin-induced nAChR cluster formation [96,97]. In addition to the promotion of presynaptic vesicle recycling [98], the effects of BDNF in the synapse are regulated by way of Wnt canonical signaling pathway [99] and activated by β2-adrenoceptor agonists (via intracellular signaling pathways, perhaps including the cyclic adenosine monophosphate, cAMP/protein kinase A/cAMP-responsive element-binding protein pathway) to maintain the structural and functional integrity of motor endplates [100]. The therapeutic pre- and postsynaptic benefits of β-adrenoceptor agonists (salbutamol and ephedrine) have been reported in MuSK MG animal models [101], neonatal myasthenia gravis (FARIS, caused by fetal AChR γ-subunit antibodies [72,73]) [102] and MG patients with nAChR antibodies [103] as well as various types of congenital myasthenic syndrome [104,105] including the patients suffering from mutations in MuSK [106] and Lrp4 [107].

Cortactin antibodies are also suggested to be involved in underlying autoimmune mechanism in MG [108,109]. Cortactin acts as a protein that is a tyrosine kinase substrate and a regulator of actin polymerization; its tyrosine phosphorylation is enhanced by agrin, suggesting that a function of phosphorylation-dependent cortactin signaling downstream from agrin/MuSK promotes actin polymerization via actin-related proteins 2/3 complex (Arp2/3 complex) and stabilizes AChR clusters at the postsynaptic membrane [40] (right part of Figure 1). Cortactin also expresses in the presynaptic side [110] and acts as a presynaptic effector molecule depending on the Wnt-signaling in Drosophila neuromuscular junction [111]. This may be worth studying in vertebrates. In fact, cortactin antibodies were found in a patient with presynaptic disease, LEMS [108].

The following synapse-related proteins may require a consideration for the synaptic organization and trans-synaptic communication although they have not been proven as immunological targets. The coronin-6 regulates AChR clustering through modulating the interaction between AChR and the actin cytoskeletal network [112]. The caveolin-3 is a MuSK kinase domain-binding protein that participates in agrin-induced phosphorylation and activation of MuSK, thereby driving nAChR clustering [113]. The microtubule-associated protein doublecortin, expressed in motor neurons and skeletal muscles, normally limits axonal growth following establishment of synaptic contact with the postsynaptic element to orderly form the pre- and postsynaptic morphology [114]. The R-spondin 2 (Rspo2), highly expressed in motor neurons and reactive with leucine-rich repeat-containing G-protain coupled receptor 5 (Lrg5, expressed in skeletal muscles and enriched in the neuromuscular junction), acts as the Wnt-dependent (via Lrp4 and MuSK) and agrin-independent regulator of nAChR clustering, and also has an effect on synaptic vesicle recycling and number of active zones in the nerve terminal [115]. This biological signal indicates that Rspo2–Lrg5 interaction plays a role in precise apposition of pre- and post-synaptic components for the synaptic transmission through the Wnt-signaling pathway [115]; the signal may be conducted via the canonical pathway including β-catenin [116]. Amyloid precursor protein (APP) and APP-like protein, well known in the pathogenesis of Alzheimer’s disease, participate in nAChR clustering via the Lrp4-MuSK signal in cooperation with agrin-mediated signal and also contribute to the presynaptic differentiation of the neuromuscular junction [117,118,119,120]. The density of ACh-containing vesicles mediated by APP at the presynaptic site is modulated by glia cell-derived neurotrophic factor (GDNF) expressed in muscle cells [121], the neuronal receptor of which is Ret tyrosine kinase [122]. APP family proteins contribute to synaptic plasticity in not only the central nervous system but also the neuromuscular junction. Shown in the central nervous system is that linking of presynaptic neurexins to postsynaptic neuroligins acts for the synaptic cell-adhesion and thereby mediates signaling across the synapse [123].

## 5. Conclusions

The present review sheds light on the molecular mechanisms that mediate the formation, stabilization and maintenance of the neuromuscular junction and trans-synaptic communication. They are based on the key molecular mediators including agrin, Wnts, Lrp4, MuSK, laminins, extracellular matrix and presynaptic receptors (including muscarinic and adenosine receptors). Insight into the functional structures organized by the synaptic and peri-synaptic proteins will foster further approach to search for new antigenic targets in immunological diseases, and also will be informative to the mutations causative of congenital myasthenic syndromes. An understanding of complex molecular mechanisms will potentially contribute to the development of target-specific therapeutic approaches to NMJ disorders.

## Figures and Tables

**Table 1:** Study of antibodies against muscle specific tyrosine kinase (MuSK) extraceullar segment (immunoglobulin-like 1 and 2 (Ig1/2) domains and cysteine-rich domain (CRD)): clinical and immunological profiles of 33 patients positive for MuSK (extracellular full-length) antibodies and negative for nicotininc acetylcholine receptor (AChR) antibodies.

Groups (Number of Patients)	10 Patients	23 Patients
Antibodies against recombinant segments	Anti-Ig1/2-positive Anti-CRD-positive	Anti-Ig1/2-positive Anti-CRD-negative
Age at onset	Age (years): 22–75	Age (years): 6–80
Gender	Female: 8/Male: 2	Female: 15/Male: 8
MuSK antibody titers determined by standard RIA (control, <0.05 nmol/L)	6.08–131.40	5.32–45.75
MG severity (MGFA grades)		
IIa	0	3
IIb	0	7
IIIa	0	2
IIIb	4	1
IVa	0	0
IVb	0	0
V	6	10
Immunoblots of purified recombinant proteins of human MuSK extracellular segments	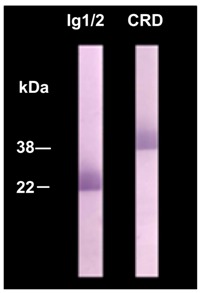	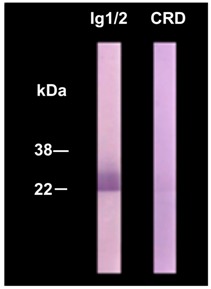

MGFA: Myasthenia Gravis Foundation of America. Figures indicate numbers of the patients subject to each item. The study collected 43 anti-AChR-negative patients, 10 of whom were negative for antibodies determined by both the standard radioimmunoassay (RIA, extracellular full-length of MuSK used as antigen) and the present study (Ig1/2 domains and CRD of MuSK used as antigens). Immunoblotting was done using purified recombinant protein of human MuSK Ig1/2 domains and CRD. Immunostained reactivity was tested with serum samples (1:500 dilution) from myasthenia gravis patients at 5 μg recombinant protein/lane; 22kDa and 38 kDa immunostained bands were visualized as anti-Ig1/2 domains and anti-CRD, respectively ; these were confirmed by using mouse anti-human monoclonal antibodies, respectively [65].
